# The Estimates of Retinal Ganglion Cell Counts Performed Better than Isolated Structure and Functional Tests for Glaucoma Diagnosis

**DOI:** 10.1155/2017/2724312

**Published:** 2017-07-24

**Authors:** Bruno L. B. Esporcatte, Andrea C. Kara-José, Luiz Alberto S. Melo, Luciano M. Pinto, Ivan M. Tavares

**Affiliations:** Glaucoma Division, Department of Ophthalmology and Visual Sciences, Escola Paulista de Medicina, Universidade Federal de São Paulo, São Paulo, SP, Brazil

## Abstract

**Purpose:**

To evaluate the diagnostic accuracy of retinal ganglion cell (RGC) counts as estimated by combining data from standard automated perimetry (SAP) and spectral domain optical coherence tomography (SD-OCT).

**Methods:**

Healthy individuals and glaucoma patients were included in this cross-sectional study. All eyes underwent 24-2 SITA SAP and structural imaging tests. RGC count estimates were obtained using a previously described algorithm, which combines estimates of RGC numbers from SAP sensitivity thresholds and SD-OCT retinal nerve fiber layer (RNFL) average thickness.

**Results:**

A total of 119 eyes were evaluated, including 75 eyes of 48 healthy individuals and 44 eyes of 29 glaucoma patients. RGC count estimates performed better than data derived from SD-OCT RNFL average thickness or SAP mean deviation alone (area under ROC curves: 0.98, 0.92, and 0.79; *P* < 0.001) for discriminating healthy from glaucomatous eyes, even in a subgroup of eyes with mild disease (0.97, 0.88, and 0.75; *P* < 0.001). There was a strong and significant correlation between estimates of RGC numbers derived from SAP and SD-OCT (*R*^2^ = 0.74; *P* < 0.001).

**Conclusion:**

RGC count estimates obtained by combined structural and functional data showed excellent diagnostic accuracy for discriminating the healthy from the glaucomatous eyes and performed better than isolated structural and functional parameters.

## 1. Introduction

Glaucoma is a neuropathy characterized by retinal ganglion cell (RGC) degeneration resulting in progressive neuroretinal rim thinning and severe excavation of the optic nerve head [1, 2]. These structural changes are often followed by functional losses that may affect vision-related quality of life [3]. Glaucoma can remain asymptomatic until the disease reaches an advanced stage [4].

The definitive diagnosis of glaucoma is based on structural changes in the optic nerve head consistent with visual field loss. However, in early stages, patients may present with structural defects in either the optic nerve head or the retinal nerve fiber layer (RNFL) that precede any visual field changes detected by standard automated perimetry (SAP) [5, 6]. On the other hand, many patients in advanced stages of glaucoma show evidence of functional deterioration, but without measurable changes in currently available structural tests [7, 8]. Therefore, the combined use of structural and functional tests would be expected to lead to an earlier diagnosis of glaucoma and a better likelihood of detection of its progression in advanced stages [9, 10].

Neither SAP nor optical coherence tomography (OCT) is able to detect RGC loss directly. Based on experimental studies in monkeys, Harwerth et al. [11] derived an empirical model relating sensitivity measurements in SAP to histological RGC density as a function of retinal eccentricity. The experimental model was then translated to clinical perimetry in humans and allowed the estimation of RGC numbers from SAP sensitivity thresholds [12]. Other formulas including the RNFL thickness measured by OCT were developed to also estimate the number of RGCs [13]. Harwerth et al. [11] demonstrated that RGC losses estimated by clinical perimetry were in close agreement with those estimated by OCT. Appropriate measurement scales for sensitivity, retinal eccentricity, and age-related neural losses were considered the key parameters for estimation of RGC losses [11].

Based on those empirical formulas, Medeiros et al. [14, 15] developed an algorithm to estimate RGC counts, which combines estimates of RGC counts from both SAP sensitivity thresholds and OCT average RNFL thickness measurements. The method includes a weighting system that provides greater emphasis to RGC estimates from OCT in early glaucoma and greater emphasis to estimates from SAP in advanced disease [13–15]. RGC count estimates derived from functional and structural tests have been shown to perform significantly better than isolated structural and functional parameters for diagnosing, staging, and monitoring the progression of glaucomatous damage [14–17].

The purpose of this study was to evaluate the glaucoma diagnostic accuracy of RGC counts as estimated by combining data from SAP sensitivity thresholds and average RNFL thickness measured by OCT. We also established a correlation between RGC estimates obtained from SAP and OCT data.

## 2. Materials and Methods

This observational, cross-sectional study was approved by the Ethical Committee of the Federal University of São Paulo and was performed in accordance with the ethical standards laid down in the Declaration of Helsinki and the International Conference on Harmonisation Guidelines for Good Clinical Practice [18]. Informed consent was obtained from all individual participants included in the study.

All subjects underwent a comprehensive ophthalmologic examination by a glaucoma specialist including review of medical history, best-corrected visual acuity, slit-lamp biomicroscopy, Goldmann applanation tonometry, gonioscopy, and dilated fundoscopic examination. Only subjects older than 40 years with open angles on gonioscopy were included. Subjects were excluded if they presented with a best-corrected visual acuity of less than 20/40 in healthy subjects or 20/80 in glaucoma patients, spherical refraction outside ±5.0 diopters and/or cylinder correction outside 3.0 diopters, or any other ocular or systemic disease that could affect the optic nerve, RNFL, or the visual field. Patients with ocular media opacities that interfered with the exams or subjects on whom cataract surgery was performed at least six months prior to the first attempt were also excluded.

Healthy subjects were recruited from the general population and were required to have a normal ophthalmologic examination, intraocular pressure below 22 mmHg, absence of optic neuropathy, and normal visual field tests in both eyes. Eyes were classified as glaucomatous if glaucomatous optic neuropathy was evident in masked grading of optic disc stereophotographs and repeatable abnormal visual field test results. All subjects that presented only one of the previous criteria were excluded from the sample.

Glaucoma patients were recruited from the Glaucoma and General Clinic at the Ophthalmology Department of the Federal University of São Paulo, Brazil. Healthy subjects were recruited from the general clinic or hospital staff.

### 2.1. Optic Disc and Posterior Pole Evaluation

Stereophotographs were obtained with the Visucam® Pro NM (Carl Zeiss Meditec, Dublin, CA, USA). Two glaucoma specialists blinded to the clinical diagnosis reviewed digital stereoscopic images with a stereoscopic viewer. In case of disagreement, a third observer served as an adjudicator. Glaucomatous optic neuropathy was defined by the presence of neuroretinal rim thinning (localized or diffused) or peripapillary RNFL defects (localized or diffused). RNFL slit defects narrower than the diameter of adjacent vessels were not included.

### 2.2. Visual Field Testing

All patients underwent SAP testing using Humphrey field analyzer (HFA-II®; Carl Zeiss Meditec, Dublin, CA, USA) and Swedish interactive thresholding algorithm standard 24-2 test less than six months apart from imaging. If a subject presented a visual field with more than 33% false-negative errors, 15% false-positive errors, or more than 20% fixation losses, this exam was excluded and the subject was rechecked. Normal visual fields were defined based on mean deviation (MD) and pattern standard deviation (PSD) within 95% confidence limits and a glaucoma hemifield test (GHT) within normal limits. An abnormal SAP test was defined as a visual field with PSD outside the 95% confidence limits or a GHT outside normal limits or a cluster of three points with a probability of less than 5% on a pattern deviation map at least one point with a probability of less than 1% in two consecutive reliable visual fields.

### 2.3. Optical Coherence Tomography

Subjects underwent ocular imaging with dilated pupils using an optical coherence tomograph, the Spectralis™ OCT (software version 5.1.3; Heidelberg Engineering, Dossenheim, Germany). The examiner was required to manually center the scan on the optic disc. To increase the image quality, the device included an automatic real-time function that gathered multiple frames, and images were averaged to reduce noise.

As previously described, infrared reflection images (*λ* = 820 nm) and OCT B-scans (*λ* = 870 nm, 40,000 A-scans/s) of the dual-laser scanning systems of the Spectralis OCT were acquired simultaneously; 3.4 mm-wide circular scans were taken (768 A-scans) around the optic disc. The average peripapillary RNFL thickness was automatically calculated by the software using a circular scan pattern with 12 degrees (3.5 to 3.6 mm) in diameter. Only exams with a signal strength of more than 15 (as suggested by the manufacturer), clear fundus image visualization, and a scan pattern without any artifacts or other algorithm failure were included in the analyses [19, 20].

### 2.4. Estimation of Retinal Ganglion Cell Number

The estimate of RGC count was based on previous works on the development and validation of a model linking structure and function in glaucoma [11, 12, 14, 15, 21, 22]. The model combines SAP and OCT measurements to provide a final estimate of RGC count in each eye.

Briefly, the first part of the algorithm obtained a SAP-derived estimate of the total number of RGCs (SAP*rgc*) by adding the estimates from all locations in the visual field, thus relating sensitivity measurements in SAP to histological RGC counts as a function of retinal eccentricity. The structural part of the model consisted in estimating the number of RGC axons from RNFL thickness measured by OCT (OCT*rgc*), considering the effect of age in RNFL, as detailed elsewhere [15, 16].

A combined measure was calculated by averaging the estimates of RGC numbers obtained from SAP and from OCT, weighted according to the severity of the disease. The following equation was used:

(1) $$\begin{array}{c} Estimated\;RGC\;count=\left(1+\frac{MD}{30}\right)\times OCTrgc+\left(\frac{-MD}{30}\right)\times SAPrgc. \end{array}$$

### 2.5. Statistical Analyses

All statistical analyses were performed with Stata software (Stata version 13; StataCorp, College Station, TX, USA). Welch's *t*-test was used to compare age, SAP MD, SAP PSD, and average RNFL thickness of unequal samples. The area under receiver operating characteristic (AROC) curves was used to compare the diagnostic accuracy of average RNFL thickness, SAP MD, and the estimated RGC count [23]. Data were adjusted for age and intereye dependency effect with bootstrapping with 10,000 replications [24]. The alpha level (type I error) was set at 0.05.

## 3. Results

A total of 119 eyes (77 participants) were evaluated, including 75 eyes of 48 healthy subjects and 44 eyes of 29 glaucoma patients. The mean age was significantly higher in glaucoma patients than in controls (68.1 ± 11.3 years versus 58.6 ± 8.8 years; *P* < 0.001). Average SAP MD was significantly lower in glaucoma patients than healthy subjects (−7.91 ± 6.81 dB versus −1.05 ± 1.05 dB; *P* < 0.001). The glaucomatous eyes had a wide range of disease severity, with SAP MD values ranging from −0.71 dB to −30.15 dB and SAP PSD ranging from 1.65 dB to 15.26 dB. Average RNFL thickness was significantly lower in glaucoma patients than in controls (73.54 ± 16.54 *μ*m versus 100.07 ± 10.01 *μ*m; *P* < 0.001) (Table 1). Healthy subjects demonstrated a significantly higher mean estimated number of RGCs than did the glaucoma group (986,396 ± 143,315 cells versus 535,479 ± 204,646 cells; *P* < 0.001).

**Figure 1:**
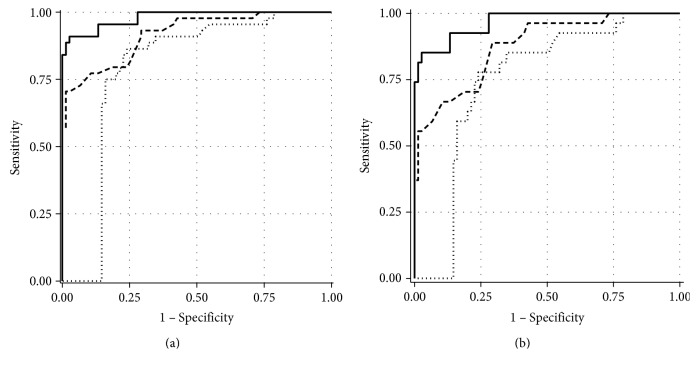
Receiver operating characteristic (ROC) curves for discriminating between the healthy and glaucomatous eyes. (a) The area under ROC curves was 0.98, 0.92, and 0.79 for estimated retinal ganglion cell (RGC) count, average retinal nerve fiber layer (RNFL) thickness, and standard automated perimetry mean deviation (SAP MD), respectively. (b) When eyes with MD < −6 dB were excluded, estimated RGC count also showed better diagnostic performance (area under ROC curves of 0.97, 0.88, and 0.75; *P* < 0.001). Black line: estimated RGC count; dashed line: average RNFL thickness; dotted line: SAP MD.

The diagnostic performance of estimated RGC count was significantly better than that of RNFL thickness measured by OCT or SAP MD for discriminating the glaucomatous eyes from the healthy eyes, with AROCs of 0.98, 0.92, and 0.79 (*P* < 0.001) (Figure 1(a)). Moreover, the estimated RGC count also presented significantly higher AROC than RNFL thickness measured by OCT or SAP MD in the eyes with MD ≥ −6 dB (0.97, 0.88, and 0.75; *P* < 0.001) (Figure 1(b)). Pairwise comparisons were performed on estimated RGC counts, RNFL thickness, and SAP MD and were determined to be statistically significant (Table 2).

However, when data were adjusted for age and intereye dependency, the difference between estimated RGC count, RNFL thickness, and SAP MD showed borderline statistical significance (AROCs of 0.95, 0.88, and 0.68, resp.; *P* = 0.05). There was no statistically significant difference between the groups in pair comparison after adjusting for age and binocularity. The diagnostic performance of estimated RGC count, RNFL thickness, and SAP MD was significantly different in the eyes with SAP MD ≥ −6 dB (0.92, 0.82, and 0.60, resp.; *P* = 0.04). Moreover, there were no significant differences in pair comparison between the three parameters (Table 2).

## 4. Discussion

In the present study, RGC count estimates based on an algorithm that combines structural and functional parameters had a better accuracy for discriminating glaucomatous from the healthy eyes than isolated SAP or OCT measurements.

The estimated RGC count in the glaucomatous eyes was lower than that in healthy subjects (535,439 ± 204,646 cells versus 986,396 ± 143,315 cells). In a cohort of patients with glaucoma in the early stage of development of visual field defects (MD = −2.17 ± 1.34), Medeiros et al. [16] observed an estimated RGC count of 652,057 ± 115,829 in the glaucomatous eyes and 910,584 ± 142,412 cells in normal subjects.

The diagnostic accuracy of the estimated RGC count was significantly better than the isolated average RNFL thickness of Spectralis OCT and SAP MD (AROCs of 0.98, 0.92, and 0.79). These results corroborate the previous study of Medeiros et al. [14], who observed that the accuracy of the estimated RGC count was higher than Cirrus™ OCT average RNFL thickness and SAP MD (AROCs of 0.95, 0.92, and 0.88). Another study also showed an accuracy of the estimated RGC count better than the Cirrus OCT RNFL thickness (AROCs 0.95 and 0.88) [16].

The estimated RGC count also presented a better performance to differentiate the healthy and glaucomatous eyes in a subgroup of patients with MD ≥ −6 dB in comparison with RNFL thickness and SAP MD (AROCs of 0.97, 0.88, and 0.75). Sihota et al. [25] observed similar AROC of RNFL thickness to separate the normal from the early glaucomatous eyes (0.90) in a protocol using Stratus™ OCT. However, the AROC was lower when this parameter was used to differentiate the early and moderate glaucomatous eyes (0.70). In a study with healthy and preperimetric eyes, Medeiros et al. [14] observed similar accuracy for RNFL thickness (0.88), but lower for SAP MD (0.63). In our study, we did not observe a considerable decrease in the AROC of the estimated RGC count when it was tested in patients with early-stage glaucoma.

In the present study, there was a significant difference in age between the glaucoma and the control groups; furthermore, some subjects had both eyes included in the sample. To minimize the effect of these variables in the analyses, a covariate adjustment was used in the AROC evaluation [26]. After the corrections, the estimated RGC count was significantly more accurate than both RNFL thickness and SAP MD with all patients included (AROCs of 0.95, 0.88, and 0.68; *P* = 0.05); similar results were obtained in the eyes with SAP MD ≥ −6 dB (0.92, 0.82, and 0.60; *P* = 0.04). However, the differences between AROCs of these three parameters showed borderline or not significant statistical difference. Even though our results are in accordance with the literature, with regard to accuracies and differences among them, the limitation that both the mean lower age of the control group and the sample size made those differences not significant. Therefore, our results ratify the value of the estimated RGC count as an important parameter to the correlation of structure and function tests in glaucoma evaluation.

There are other methods to estimate the number of RGCs in the human retina. Recently, Raza and Hood [27] developed one such method in which the RGC density was obtained from the literature and the thickness of macular RGC layer was measured directly with a swept-source (SS) OCT [28]. The mean estimated RGC number presented a good agreement with estimates of visual sensitivity and histological counts. Moreover, the studied parameter showed a lower variance when compared to the RGC count obtained with Harwerth's algorithm [11]. Unfortunately, we could not include it in our study because we did not have an SS-OCT available at the time of the study, and the Spectralis OCT software used in this study did not have macular or optic disc analyses.

There were some limitations in the present study. Empirically derived formulas to estimate the number of RGCs from SAP and OCT data and the estimates of RGC count were not based on direct histologic RGC counts in humans. However, the formulas have been validated on histological studies in monkeys and applied to multiple external cohorts in humans [11, 16] Other limitations were a small sample size, a limited number of eyes in different stages of glaucoma, the use of multiple comparisons, and the age difference between the healthy and glaucoma groups. Moreover, the definition of glaucoma through the structural evaluation of the optical disc and RNFL defects could overestimate the diagnostic accuracy of the structural measure (average RNFL thickness) OCT regarding the accuracy of the functional measure from SAP.

There was a significant strong correlation between RGC estimates obtained from SAP and Spectralis OCT data. The estimated count of RGCs obtained by combining the RNFL thickness of Spectralis OCT with SAP sensitivity showed excellent diagnostic accuracy for discriminating between the normal and glaucomatous eyes, even in earlier stages of the disease. This accuracy was better than the structural and functional parameters isolated; however, after adjusting for age and dependency between the eyes, the differences between these parameters showed borderline or no statistical significance. Furthermore, when analyzing the early-stage glaucoma subgroup, the differences between the accuracies were statistically significant.

Finally, the present study corroborates previous published results but using a different OCT device (Spectralis OCT); moreover, it replicates the use of the estimated RGC count algorithm in an independent study population with a different racial composition. The purposed algorithm is an important diagnostic tool that mixes structural and functional parameters for glaucoma diagnosis and that also seems to have a great potential to be used for follow-up.

## Figures and Tables

**Table 1:** Demographic and clinical characteristics of the study participants (*n* = 119).

	Healthy subjects (*n* = 75)		Glaucoma patients (*n* = 44)	
	Mean ± SD	Range	Mean ± SD	Range
Age (years)	58.6 ± 8.8	44.6 to 77.9	68.1 ± 11.3^∗^	44.7 to 85.6
SAP MD (dB)	−1.05 ± 1.05	−3.57 to 1.42	−7.91 ± 6.81^∗^	−30.15 to −0.71
SAP PSD (dB)	1.52 ± 0.22	1.10 to 1.98	6.56 ± 4.55^∗^	1.65 to 15.26
RNFL thickness (*μ*m)	100.07 ± 10.01	80 to 128	73.54 ± 16.54^∗^	39 to 107
Estimated RGC count	986,396 ± 143,315	722,128 to 1,381,549	535,479 ± 204,646^∗^	34,128 to 889,775

SAP MD: standard automated perimetry mean deviation; SAP PSD: standard automated perimetry pattern standard deviation; RNFL: retinal nerve fiber layer; RGC: retinal ganglion cell. ^∗^*P* < 0.001, healthy subjects versus glaucoma patients. Welch's *t*-test was used to compare age, SAP MD, SAP PSD, and average RNFL thickness of unequal samples.

**Table 2:** Differences between unadjusted data and data adjusted for age and binocularity.

	*P* value	
	Unadjusted data	Adjusted data^a^
*All eyes*		
Estimated RGC count versus RNFL versus SAP MD	<0.001	0.05
Estimated RGC count versus RNFL	0.004	0.06
Estimated RGC count versus SAP MD	<0.001	0.08
RNFL versus SAP MD	0.007	0.19
*MD ≥ −6 dB*		
Estimated RGC count versus RNFL versus SAP MD	<0.001	0.04
Estimated RGC count versus RNFL	0.005	0.08
Estimated RGC count versus SAP MD	<0.001	0.06
RNFL versus SAP MD	0.03	0.19

SAP MD: standard automated perimetry mean deviation; RNFL: retinal nerve fiber layer thickness (average value); RGC: retinal ganglion cell. ^a^Adjusted for age and binocularity.
